# Editorial: Use of behavioral metrics and measures in government

**DOI:** 10.3389/frma.2023.1172748

**Published:** 2023-03-22

**Authors:** Richard Aragon, Priscilla W. Clark

**Affiliations:** ^1^National Institute of General Medical Sciences, United States National Institutes of Health, Bethesda, MD, United States; ^2^United States Department of Housing and Urban Development, Washington, DC, United States

**Keywords:** public sector, government, operations, behavioral metrics, organizational performance

Public sector organizations throughout the United States and the world have continually attempted to derive and apply various metrics and measures to assess and improve their ability to deliver on their respective missions. Within the federal government of the United States, for instance, traditional metrics such those related to organizational performance measurement have been codified into law and are continually required of all federal agencies (United States Congress, 1993, 2010). These metrics and measures have been supplemented by a newer generation of parameters that aim to measure and capture data and information related to various aspects of organizational performance and impact at both the federal and state levels (Performance.Gov, 2023; State of Maryland Department of Budget and Management, 2023).

Despite the recent explosion in the number of metrics and measures used to assess public sector organizational efficiency, one area of measurement and analytics remains comparatively under-explored and undocumented: the application and use of behavioral metrics. This area of assessment represents the most important of all because it attempts to consider, describe, and measure (if not *predict*) the impact of human behaviors on varying aspects of an institution or organization's operations.

The Federal Employee Viewpoint Survey (FEVS), for instance, is an instrument that is administered by the U.S. Office of Personnel Management (OPM) to acquire data and information on employee *perceptions* and *perspectives* regarding a myriad of parameters related to government operations (United States Office of Personnel Management, 2023). Such parameters include, but are not restricted to, innovation, workload, effective leadership, and the performance of federal agencies relative to one another (Partnership for Public Service, 2021). FEVS thus measures employees' *perceptions* about the organizational and *behavioral* factors that influence recruitment, retention, leadership, and job satisfaction. It is through these specific parameters that the ability of an agency to meet its mission is both measured and evolved (changed). FEVS further provides employees with the opportunity to influence this type of change from within their own agencies by submitting feedback about individual work environment, exposure to leadership, and similar aspects of organizational function. The survey itself is required by law (United States Congress, 2003). The United States Congress established a requirement for agencies to conduct this annual survey of federal employees to assess workforce satisfaction as well as leadership and management practices that contribute to agency success and performance. The commercial sector has leveraged and functionalized behavioral metrics and measures in a predictive sense for a much longer period than public sector organizations, as evidenced by the ongoing ability of companies to continually filter content and present web information based on an individual user's browsing and/or purchase (i.e., behavioral) history.

The application of behavioral metrics has not occurred without controversy. In 2018, UK-based Cambridge Analytica became involved in a protracted series of highly publicized political and legal battles that resulted in the company's eventual demise (New York Times, 2018). In this specific case, procedures regarding informed consent were either lacking or non-existent and ultimately led to the creation and use of unauthorized “psychographic” profiles (Bakir, 2020). These examples illustrate the expanding use of behavioral metrics and measures as forms of analytics within the commercial arena in the former case and within the political arena in the latter case.

To develop an *initial* evidence base from which to better understand the utility of behavioral metrics in public sector institutions and organizations (e.g., governments and universities), this topic area presents four original articles that illustrate the varying ways in which behavioral metrics are utilized, both within the United States federal government and at an international institution of higher education. The article by Keckler, for instance, considers the joint effects of organizational performance data and measures of *organizational independence* on programmatic outcomes. Similarly, the perspectives piece by Miller examines the role of an organization's *risk culture* on programmatic decisions and then utilizes this data to positively evolve both risk management controls and individual institutional training paradigms. Leveraging and extending this theme of culture, Goon et al. considers and assesses how the documented *everyday experiences of federal employees*, particularly those of Asian American, Native Hawaiian, and Pacific Islander (AANHPI) descent, can be combined with analytic practices to better understand and address historical—if not persistent—inequities in both inclusion and opportunity. Finally, the article by Hashiguchi et al. examines the association between the research productivity or output of promising, next generation researchers and the *past research activities and behaviors* of their supervisors.

As illustrated by the above examples, the collective aim of this topic area is not only to introduce the concept of behavioral metrics as an adjuvant to the measurement of organizational performance but also to its improvement. Because organizations are composed of people, developing the capacity and means to better understand and measure individual behaviors is critical to understanding and ultimately improving organizational performance. The importance of this fact becomes evident when one considers the increasing diversity and number of generations in the current workforce (Hyman et al., 2022). Thus, it is our hope that these initial studies will serve as the impetus for the careful consideration and application of behavioral metrics as well as the identification of best practices for their use within public sector environments.

## Author contributions

Both authors listed have made a substantial, direct, and intellectual contribution to the work and approved it for publication.
